# An assessment of the utility and repeatability of the renal resistive index in horses

**DOI:** 10.1371/journal.pone.0226941

**Published:** 2019-12-26

**Authors:** Natalia Siwinska, Agnieszka Zak, Malwina Slowikowska, Barbara Szczepankiewicz, Artur Niedzwiedz, Urszula Paslawska

**Affiliations:** Department of Internal Medicine and Clinic of Diseases of Horses, Dogs and Cats, Faculty of Veterinary Medicine, University of Environmental and Life Sciences, Pl. Grunwaldzki, Wroclaw, Poland; University of Bologna, ITALY

## Abstract

The aim of this study was to establish the value of the renal resistive index (RI) of intrarenal arteries in healthy warmblood non-racing horses of different ages to assess the influence of physiologic factors and repeatability of measurement. The kidney ultrasonography examination was performed in three age groups: 15 foals, 15 adults and 15 elderly horses. The procedure was performed in unsedated standing animals. RI values were measured using pulsed-wave Doppler at the medial part of each kidney in the intrarenal arteries. To evaluate repeatability, all measurements were repeated two hours after the first examination. Statistical analysis of the relationship between groups was carried out using Fisher’s test. The relationship between the RI value and the physiological parameters was evaluated using linear regression. Repeatability of measurements was determined based on the Bland-Altman plot. The mean RI value in the studied horses was 0.48 ± 0.05 in the left kidney and 0.49 ± 0.05 in the right kidney. There were no statistically significant differences between the RI values in foals and adult horses. The elderly horses had a significantly higher RI value. Pulse pressure was the only physiological parameter affecting the RI value. The repeatability coefficient was 0.089 for the right kidney and 0.09 for the left kidney. The presented result suggest that elderly healthy horses have higher RI values than younger animals, which should be taken into account in clinical practice. The arterial pulse pressure should also be considered when interpreting RI values. The measurements have high repeatability, but in the authors’ opinion, this procedure is time consuming and requires experience.

## Introduction

Numerous studies carried out on humans and small animals have demonstrated the potential use of several Doppler ultrasound parameters to improve clinical diagnostics of the kidney status [1]. One of them includes the resistive index (RI), a physiological parameter that indirectly reflects the degree of intraorgan vascular resistance, enabling the detection of kidney pathologies associated with renal vascular resistance [2]. An increase in the kidney vascular resistance is considered an important marker of kidney damage irrespective of its cause, i.e. inflammatory disease, renal artery stenosis, obstructive renal disease, acute kidney injury or chronic kidney failure [3–7]. In humans, the RI has become a valuable tool used to monitor the response to therapy and is used in transplantology. In human medicine, factors that affect the RI include age, blood pressure, pulse and respiratory rate [5,8]. In contrast to the number of human RI studies, there are only two studies assessing Doppler ultrasonography in intrarenal vessels in horses [9,10]. Unfortunately, the groups of animals were very small in both studies and consisted of only Thoroughbred horses. This limits the detailed analysis of the factors that influence RI in horses. The objective of the study was to assess: 1) the normal value of the renal resistive index of intrarenal arteries in healthy warmblood non-racing horses, 2) the impact of age, body mass, heart rate and blood pressure on the intrarenal arteries resistive index, 3) differences in the RI between the left and right kidney, 4) measurement repeatability.

## Materials and methods

### Animals and examination

**Fig 1:**
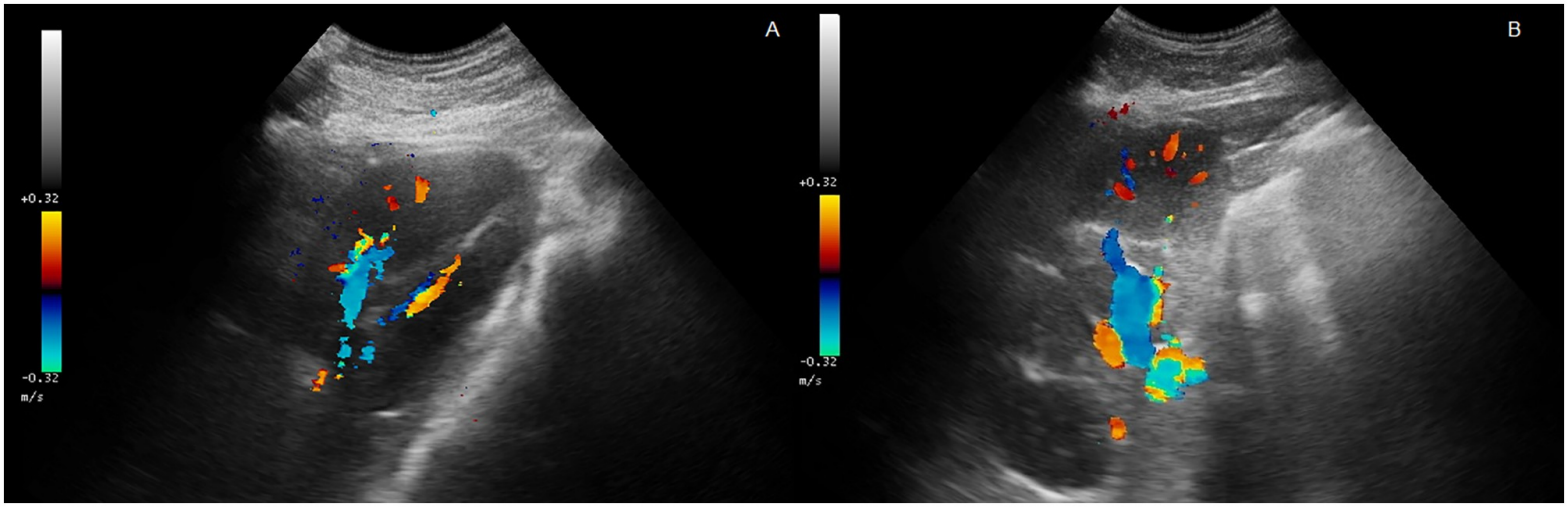
Colour Doppler ultrasound image showing the intrarenal vessels in the right kidney of a foal (A) and an adult horse (B).

Forty-five non-racing healthy horses of various breeds (the most popular breeds used in Poland: 18 Silesian horses, seven Polish warmblood cross-breed horses, five Hanoverian horses, 5 Holstein horses, four Malopolski horses, four Wielkopolski horses, two German warmblood horses), including 15 foals up to 6 months old (mean 4.3 ± standard deviation [SD] 1.6 months old), 15 adult horses from 4–10 years old (7.6 ± 3.6 years old) and 15 elderly horses above 18 years old (21.2 ± 2.65) were included in the study. The group included 23 non-pregnant females and 22 males (11 geldings and 11 stallions). The adult horses were used for low-level sport (jumping and dressage regional competitions), pleasure and some were retired. The animals belonged to private owners and were kept in three centres in Lower Silesia (GPS 50.843764, 16.299035, 400m altitude and 51.230245, 16.348189, 200m altitude) and Wielkopolska (GPS 52.510620, 16.366603, 100m altitude). The animals were kept in individual stalls apart from foals, which were kept in larger, partitioned stalls with their mothers. All the animals had daily access to pasture with green grass and working horses trained four times per week. The horses were fed roughage consisting of hay available in nets *ad libitum* and concentrate feed consisting of oats, commercial basic muesli or muesli for lactating mares fed three times a day according to the feeding dose based on the horse’s individual body weight. Foals were fed milk by their mothers but also had access to concentrate feed and roughage. All the animals had access to clean water. The mass and wither height of each animal were assessed using a girth tape. The horses were classified as healthy based on a thorough anamnesis provided by the owner/carer, a full clinical examination, a complete blood count, blood biochemistry (serum creatinine, urea, aspartate aminotransferase, alanine aminotransferase, γ-glutamyl transpeptidase, alkaline phosphatase, creatine kinase, total protein, albumin, glucose, sodium, potassium, chlorine, magnesium, calcium) and a urinary analysis (complete urinalysis with sediment examination). The horses were enrolled in the study based on the following inclusion criteria: no history of urinary or cardiovascular disease, no current symptoms of disease, no ultrasound changes in the kidneys, no drug administration in the 6-month period prior to the study. Foals were included if they were delivered by healthy mares that had a normal gestation, parturition and placenta. The horses were examined without pharmacological sedation or restraint as it could have influenced cardiovascular parameters. All the horses selected for the examination previously received basic veterinary care, including blood pressure measurements and a transabdominal ultrasound. Imprint training was commenced on the foals from their first day of life. In order to limit stress, the horses were examined in known surroundings–in their own stalls or in the stable corridor or in front of their stall. The foals were examined in the presence of their mothers in their stalls. The blood pressure was assessed non-invasively using a Mindray PM-600Vet device (oscillometric method). The cuff was placed on the tail and five subsequent measurements (including SAP—systolic arterial pressure, DAP—diastolic arterial pressure, MAP–mean arterial pressure) were collected from the medial coccygeal artery. The mean pulse pressure (PP) was calculated from the SAP and DAP using the following formula: PP = SAP–DAP. All the animals also underwent an echocardiographic examination with a simultaneous ECG recording using MyLab ^™^ 30Gold Vet (Esoate) equipped with a 2.5–5 MHz sector probe (Esoate) in order to exclude heart disease. The kidney ultrasound was carried out with the same ultrasound equipment and a 1–5 MHZ convex probe (Esoate). The first kidney ultrasound examinations were performed in the morning (8:00–11:00 am). The fur in the paralumbar area was clipped for the transabdominal kidney ultrasound examination (the 16–17 ICS on the left, the 15–17 ICS on the right) with an electric clipper and blade no. 40. The skin was thoroughly washed with water and soap containing chlorhexidine. It was then flushed with alcohol to degrease it, dried and covered by a water-soluble coupling gel. Prior to the Doppler ultrasound, the structure, echogenicity and size of the kidneys were assessed using gray-scale ultrasonography. The probe placement, frequency and other image parameters were altered depending on the amount of fat tissue and the depth of the kidney location in order to optimise image quality. After imaging the kidneys in a sagittal or parasagittal plane (anatomical planes described by Hoffmann *et al*. [11]), the intrarenal arteries were localised using the colour Doppler technique (Fig 1).

The flow spectrum was obtained using the pulsed-wave Doppler system. The sample volume size was set at 5 to 10 mm and decreased to the smallest possible size. A wall filter of 50 or 100 Hz was used to eliminate low-frequency Doppler shifts. RI values were measured at the medial part of the kidney in three kidney vessels until three adequate consecutive spectral waveforms were obtained. The RI was calculated based on the ratio between the peak systolic velocity (PSV) and end diastolic velocity (EDV), according to the following formula: RI = (PSV-EDV)/PSV [12]. It was measured automatically by the ultrasound machine software. The mean RI value for each kidney was calculated from three measurements obtained from intrarenal arteries—the interlobular or arcuate arteries. In those animals where all the RI measurements were obtained from both kidneys, a second ultrasound was repeated two hours after the first measurements. All the measurements were carried out by one researcher and experienced ultrasonographer (NS), who holds a license to perform studies on animals (number 105/2016). Hard copies of two-dimensional, colour Doppler and pulsed-wave Doppler images were saved and stored as DICOM images. All of the examinations performed in this study were non-invasive and are routinely performed in everyday medical practice. In accordance with the existing law applicable in Poland, Experiments on Animals Act from January 15^th^ 2015 (Journal of Laws of the Republic of Poland, 2015, item. 266), all animal experimental procedures in this study were performed with the approval of the 2nd Local Ethics Committee on Animal Experimentation in Wroclaw (permission No 86/2018). The study was performed on privately owned horses, hence the owners provided informed consent for the clinical research and publication of the results.

### Statistical analysis

Quantitative variables were presented as mean, standard deviation, median and quartiles (due to a non-normal data distribution), while quantitative data was assessed using abundance and percent fractions. Quantitative data between the age groups were compared using the Kruskal-Wallis test with the Conover post-hoc analysis and Holm’s correction. The independence of individual qualitative variables between the age groups was calculated using Fisher’s test. The relationship between the RI value and the remaining parameters was evaluated using one-way (right kidney/left kidney) and multifactorial (foals/adults/elderly) linear regression. Repeatability was determined based on the Bland-Altman plot with a coefficient of repeatability and by comparing the significance of the differences obtained in the Student t-test. The analysis was carried out using R for Windows (version 3.4.4).

## Results

Mean values of all clinical data obtained from examined horses are summarized in Table 1.

**Table 1:** Mean values with standard deviation of body mass, height, pulse frequency, pulse pressure and mean arterial pressure in examined foals, adults and elderly horses.

Parameters	Foals n = 15	Adults n = 15	Elderly n = 15
Body mass kg	205.1 ± 38.8*^	558.4 ± 95.3	542.6 ± 74.6
Body high cm	112.8 ± 13.6*^	161.9 ± 13.9	163.3 ± 8.9
Pulse/min	63.9 ± 6.5*^	37.1 ± 3.4	38.7 ± 4.9
PP mmHg	50.3 ± 6.4*	37.8 ± 7.9	46.2 ± 10.8#
MAP mmHg	88.8 ± 9.5*	74.8 ± 9.3	78.5 ± 10.7

*Note*: n–number of animals in the group, PP–pulse pressure, MAP–mean arterial pressure;

* statistical significance p<0.05 between foals and adults;

^ statistically significant differences p<0.05 between foals and elderly horses;

^#^ statistically significant differences p<0.05 between adult and elderly horses.

The mean age in the studied groups differed significantly (p<0.002). The body mass and height of the foals was significantly lower (p<0.001) while the mean pulse and blood pressure was significantly higher (p<0.05) than that of the adult and elderly horses. No significant differences in the body mass, height and blood pressure were noted between adult and elderly horses. The PP value was significantly higher in foals (p = 0.0006) and elderly horses (p = 0.006) compared to adult horses.

The renal RI was acquired in all the horses. The left kidney RI values proved too difficult to obtain from one foal due to lack of cooperation of the animal during the examination. In some animals, the pulsed-wave Doppler registration was difficult and time consuming due to difficulties in optimalisation of the ultrasound images. It was more difficult to obtain good quality recordings from the left kidney than from the right kidney. The examination time was elongated in the case of body motion during the ultrasound examination. Due to body movements, only two Doppler flow measurements were obtained in two adult horses and three elderly horses. In total, 111 RI values were measured in the left kidney and 135 RI values were measured in the right kidney. The results are presented in Table 2.

**Table 2:** Mean values of resistive index of the intrarenal arteries in the studied horses.

Group	RI Left kidney						RI Right kidney					
	min	max	Mean ± SD	q1	q3	Median	min	max	Mean ± SD	q1	q3	Median
**Foals**	0.38	0.53	0.46 ± 0.04	0.44	0.49	0.46*	0.37	0.55	0.47 ± 0.05	0.44	0.51	0.48*
**F**	0.41	0.53	0.46 ± 0.03	0.44	0.53	0.46*	0.37	0.55	0.47 ± 0.05	0.44	0.49	0.48*
**M**	0.38	0.50	0.45 ± 0.04	0.40	0.49	0.47*	0.38	0.55	0.48 ± 0.05	0.44	0.52	0.48*
**Adults**	0.38	0.58	0.48 ± 0.05	0.45	0.51	0.47^	0.40	0.60	0.48 ± 0.05	0.45	0.52	0.48^
**F**	0.38	0.58	0.49 ± 0.05	0.46	0.52	0.48^	0.41	0.60	0.49 ± 0.04	0.46	0.53	0.49^
**M**	0.39	0.57	0.47 ± 0.04	0.44	0.50	0.47^	0.40	0.58	0.47 ± 0.05	0.44	0.50	0.47^
**Elderly**	0.41	0.63	0.52 ± 0.05	0.49	0.57	0.53*^	0.41	0.62	0.52 ± 0.05	0.49	0.56	0.52*^
**F**	0.44	0.59	0.53 ± 0.04	0.50	0.57	0.51*^	0.41	0.62	0.53 ± 0.06	0.50	0.56	0.52*^
**M**	0.41	0.63	0.52 ± 0.07	0.46	0.56	0.54*^	0.42	0.60	0.52 ± 0.05	0.46	0.55	0.52*^
**All F**	0.38	0.59	0.49 ± 0.05	0.45	0.52	0.49	0.37	0.62	0.49 ± 0.05	0.47	0.53	0.49
**All M**	0.38	0.63	0.48 ± 0.05	0.44	0.51	0.47	0.38	0.60	0.49 ± 0.05	0.45	0.52	0.48
**All**	0.38	0.63	0.48 ± 0.05	0.45	0.51	0.48	0.37	0.62	0.49 ± 0.05	0.45	0.53	0.49

*Note*: RI–resistive index, F–female, M–male, min.–minimal values, max.–maximal values, mean ± SD–mean value with standard deviation, q1 –first quartile, q3 –third quartile.

* statistical significance between foals/elderly horses p<0.05;

^ statistical significance between adult horses / elderly horses p<0.05.

**Fig 2:**
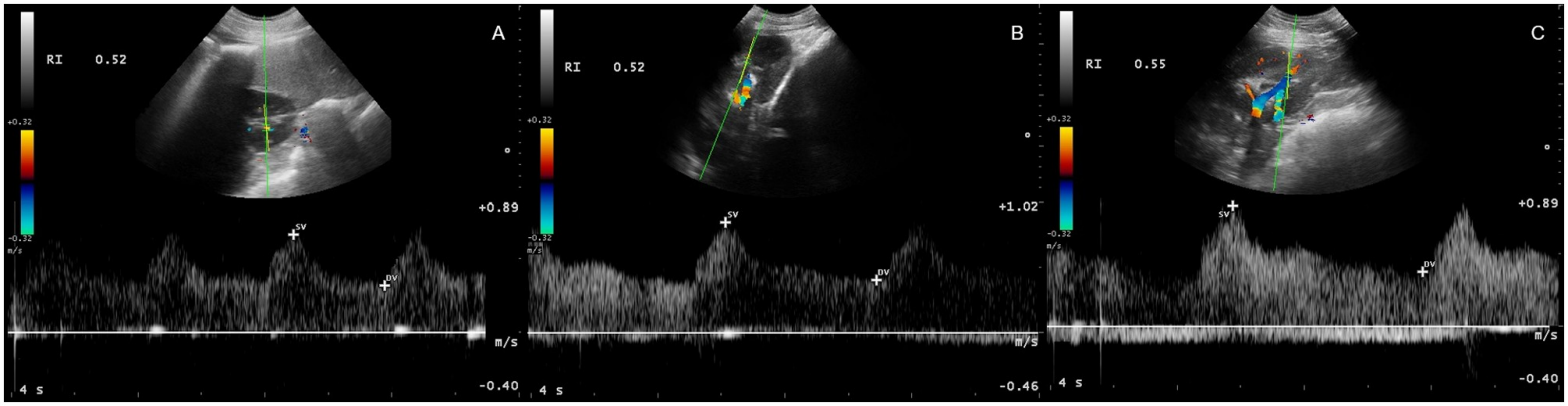
The pulsed-wave Doppler flow and resistive index measurement of intrarenal vessel of (A) foal, (B) adult horse and (C) elderly horse.

The mean RI value in the studied horses was 0.48 ± 0.05 in the left kidney and 0.49 ± 0.05 in the right kidney. In the group of foals, the mean RI value was 0.46 ± 0.04 in the left kidney and 0.47 ± 0.05 in the right kidney. The mean RI value was the same in both kidneys in the adult and elderly horses and amounted to 0.48 ± 0.05 and 0.52 ± 0.05, respectively. There were no statistically significant differences between the RI values in foals and adult horses (left kidney p = 0.1036, right kidney p = 0.5454). The elderly horses had a significantly higher RI value compared to foals (left kidney p = 0.0002, right kidney p = 0.0024) and adult horses (left kidney p = 0.0145, right kidney p = 0.0089)(Fig 2).

The lowest RI value in all the studied horses was 0.37, which was also the lowest value in the group of females. The highest RI value was 0.63 and was the highest value in the males. The lowest value in male horses was 0.38, while the highest value in female horses was 0.62. The lowest RI value in all the animals was recorded in the left kidney, while the highest value was measured in the right kidney. The maximal difference between the measurements obtained from one kidney was 0.03, while the maximal inter-kidney differences was 0.09 in an individual animal. These values indicate a narrow RI reference range in horses independent of their age and sex.

The analysis of the correlation between the RI values indicated that they were positively affected by the PP values (r = 0.61, p = 0.021 in foals, r = 0.51, p = 0.53 in adult and elderly horses). Other factors such as age (left kidney p = 0.29, right kidney p = 0.75), body mass (left kidney r = 0.17, p = 0.26, right kidney r = 0.07, p = 0.69), wither height (left kidney r = 0.32, p = 0.035, right kidney r = 0.2, p = 0.19), pulse (left kidney r = -0.17, p = 0.26, right kidney r = -0.24, p = 0.12) or the mean arterial pressure (left kidney r = -0.09, p = 0.57, right kidney r = -0.19, p = 0.19) did not affect the RI values. There were no statistically significant differences between the measurements in the left and the right kidneys (p = 0.42).

**Fig 3:**
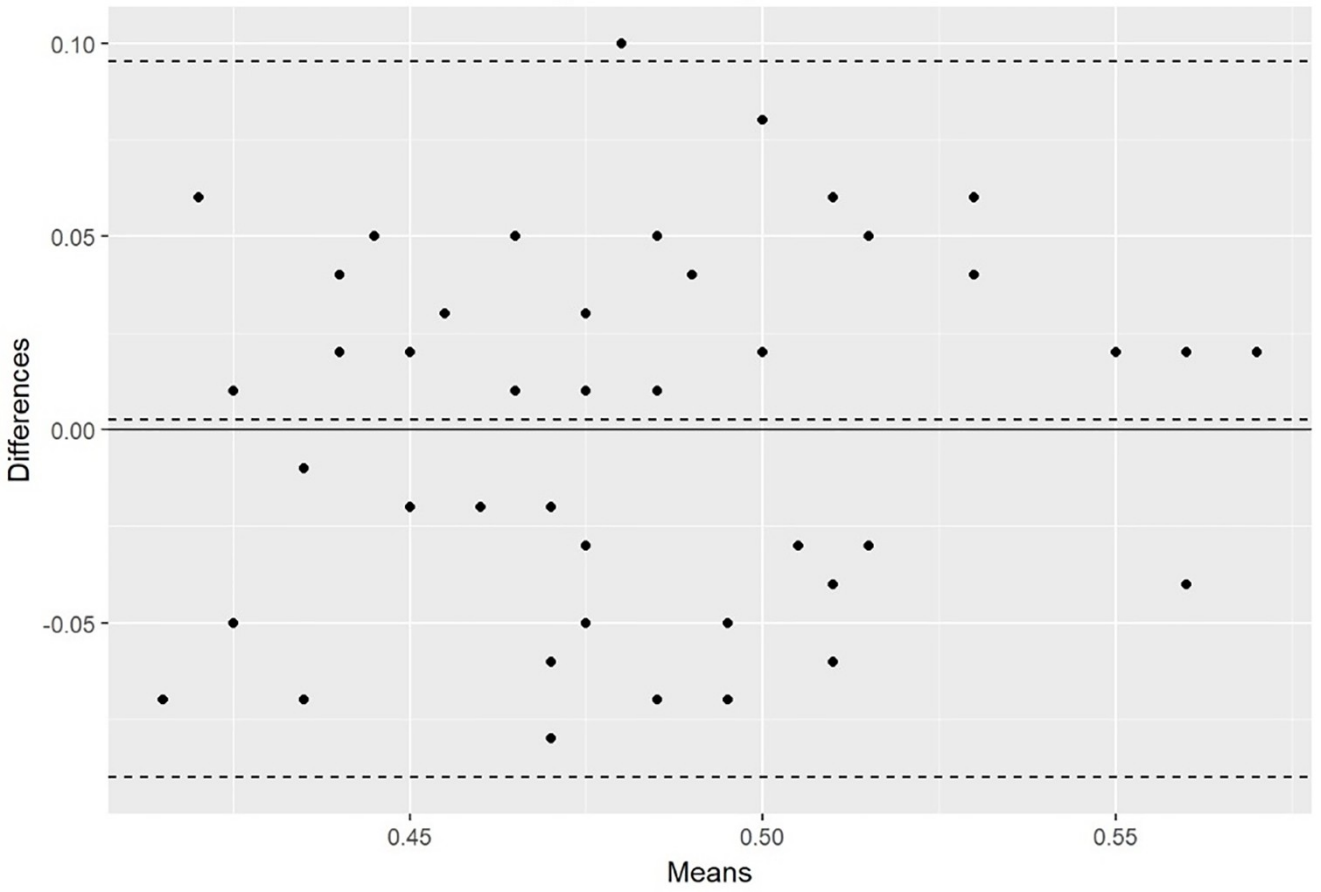
Blan-Altman plot showing the coefficient repeatability of RI measurements in examined horses.

High repeatability of the RI measurements in the intrarenal arteries was noted: the repeatability coefficient was 0.089 for the right kidney and 0.09 for the left kidney (Fig 3).

## Discussion

According to the authors’ best knowledge, these are the first findings documenting the influence of physiological parameters on the RI values in healthy non-racing horses of different ages and of both sexes. One of the key features of this study was determining the RI reference range for adult horses as well as foals and elderly horses.

Doppler ultrasonography seems to be an attractive diagnostic tool as it enables wide, non-invasive, pain-free imaging that can be carried out in all patients as frequently as needed. Doppler ultrasonography of blood flow in horses is often carried out as part of the cardiological examination and increasingly frequently in reproduction and orthopaedics [13–15]. This has led to the development of equine reference values. However, this technique has not been clinically used to assess equine kidneys. The RI is a common Doppler index used to assess normal kidney function in humans and small animals as it is considered the most consistent intrarenal Doppler parameter [16].

According to numerous studies carried out in cats, dogs, sheeps and humans, the RI values below 0.70 have been found to be normal, while increased RI values indicated renal pathology [17–22]. Furthermore, RI seems to be independent of the filtration rate because it does not change before and after furosemide administration [9]. Hoffman *et al*. established that the mean arcuate artery RI values in sedated horses were 0.549 ± 0.044, and the values for the pyelorenal artery were 0.512 ± 0.004 [11]. The RI values obtained in our study are, to mostly consistent with the results of Hoffman *et al*.

Marci *et al*. showed differences between RI in the left (0.52 ± 0.04) and right kidney (0.48 ± 0.06) in trained as well as in non-trained Thoroughbred horses (left 0.55 ± 0.01 and right 0.58 ± 0.06 kidney, respectively) [10]. The higher RI level in the right kidney was assumed to be caused by the larger size and volume of the right kidney compared to the left kidney. In healthy humans and cats, statistically significant differences in the RI values were observed between the right and the left kidney [12,23]. This was explained by a possible bias of the ultrasonographer or the presence of asymptomatic lesions in the kidney, which are not detectable in the clinical examination in the standard ultrasound examination or in laboratory tests [12]. In contrast to those studies, the results obtained in other studies on cats and dogs did not reveal significant differences in the RI values between the left and the right kidney [5,24,25]. Similarly, no differences in the RI between the left and right kidney in healthy horses were found in our study. Breed predispositions to RI differences in Thoroughbred horses cannot be excluded.

The analysis of the age groups revealed that the RI values were similar between foals and adult horses. In humans, RI values were found to be increased in newborn infants and young children [26,27], similarly to dogs, where the RI was significantly higher in very young animals compared to older ones [5]. Such a phenomenon was not observed in other animal species. According to reports in human medicine, the RI value decreases with age and reaches adult values in 8–10 year olds [3]. The RI values in the studied foals were similar to the values obtained in adult horses, which may be associated with the fact that the foals were not newborns (the mean age of the studied foals was 4.3 ± 1.6 months). Hence, this phenomenon cannot be excluded in horses. To date, there has been no data concerning the RI values in newborn foals and the age at which RI decreases to adult reference values. In humans, vascular blood flow parameters also change with aging [1,28]. A positive correlation between the RI values and age was found in a group of 147 people from 12 to 92 years old [1]. In the study by Ponte *et al*., a sudden increase in the RI was observed in people above 40 years old [28]. In the studied elderly horses, a significantly higher RI mean value compared to foals and adult horses was observed. The increased RI value is attributed to poorer kidney function due to senile changes such as arteriosclerosis or fibrosis, which may be associated with hypertension and/or intraparenchymal arteriolar damage and glomerular sclerosis [1,29]. Kidney function, including the glomerular filtration rate, renal plasma flow and urine concentrating capacity is known to decrease with age, similarly to the secretion of hormones, such as the antidiuretic hormone or renin [1]. An increase in the RI value may not only be associated with senile body changes but also with asymptomatic organ damage, although an association between glomerular, tubular or interstitial histological changes and the RI values has not been confirmed [30].

In this study in all age groups, there was no difference in the RI value between horses of different sexes. This result is consistent with previous findings assessing the RI in 5 geldings and 8 mares [10] and the study of Tetsuka *et al*. in humans [1]. In contrast, the study by Ponte *et al*. carried out on more than 700 people found significantly higher RI values in women compared to men. That result may have been caused by an older age of the female study participants [28].

It is commonly known that changes in arterial blood pressure affect the RI values [2]. Peripheral resistance is the main indicator of constant blood pressure (or the mean arterial pressure) [31]. In the present study, no correlation between the RI value and arterial blood pressure was found, which corresponds to the findings in dogs [5]. This may be due to low measurement accuracy of the oscillometric machine. The direct measurement of arterial blood pressure in horses is more reliable although it cannot be carried out in unsedated standing horses. HR is another factor that may influence the RI [30]. In humans, higher HR values were found to decrease the RI value by increasing the end-diastolic velocity [28]. On the other hand, a sudden decrease in pulse causes an increase in the RI [32]. Such findings suggest that the RI values should be interpreted cautiously in animals with an abnormal or increased pulse. In the present study, such a relationship was not detected. This may be due to the small differences in pulse values in the studied healthy horses, which did not suffer from bradycardia, tachycardia or arrhythmia. However, an association between the RI values and the pulse pressure, reported in other studies, was confirmed [30,33].

The RI value is independent of the angle and position of the exploratory probe, which limits measurement errors [12]. RI is superior to absolute flow velocity as the angle of insonation does not have to be known, and it enables the assessment of small or sinuous vessels [24]. Factors that affect the kidney ultrasound parameters include the breathing movements, and the interference of other organs, such as the ribs, spleen, intestine or lungs with the ultrasound waves [18]. It was difficult to eliminate patient movement in this study, as the animals shifted their weight from one leg to the other or their skin was twitching. The studied horses did not receive pharmacological agents due to the documented effect of such drugs on blood pressure and RI values (increase or decrease of RI values depending on the substance) [18,25]. The shape of the Doppler wave and its values may also change under stress [19]. An attempt was made to minimalise stress by carrying out the examination in the surroundings that were familiar to the horses, as well as by giving the animals time to get used to the examination technique. The measurements were recorded only after the horses were calmed down.

The reliability and specificity of the measurement of the intrarenal arterial vascular resistance in humans is controversial [34]. In some research the Doppler examination is considered accurate and reproductible [28,35]. Mikkonen *et al*. describes poor repeatability of intrarenal Doppler measurements in healthy patients [16]. Rawashdeh *et al*. came to a contrary conclusions when they found high reproducibility of RI measurements obtained from pigs at short intervals [2]. However, that study was carried out on animals under general anaesthesia. Presented study investigated intraobserver variation (repeatability) of the intrarenal RI with particular emphasis on the short-term aspect of such measurements. According to our study, short-term repeatability of the RI in horses was high in both kidneys. In order to exclude operator-related variability, patient variability and equipment variability, all the measurements were taken using the same ultrasound machine, by the same experienced examiner, in similar conditions, from the animals whose previously obtained kidney images and RI measurements were of good quality.

RI measurements were not obtained from certain animals in the study, particularly from the left kidney. This was caused by limited transabdominal imaging of the left kidney. In horses, left kidney imaging may be difficult due to intestinal gas from the small and large colon, which can restrict visualization [11,36,37]. Due to the fact that the left kidney is located under the spleen, the depth of imaging is increased by using low frequency ultrasound, which reduces the image resolution. Hoffman *et al*. reported similar limitations of the Doppler flow measurement in the left kidney [9]. It was also more difficult to obtain reliable RI values in the left kidney in animals with a thicker layer of subcutaneous tissue and well-developed muscles due to limitations in acquiring satisfactory Doppler signals from deep locations.

It was not possible to carry out a biopsy and histopathological examination of the renal tissue to exclude kidney disease, which was a limitation of this study. The owners of the examined horses did not consent to kidney biopsy due to the risk of complications (particularly in the case of a bilateral kidney biopsy). Transrectal ultrasonography of the kidneys was not performed due to anatomical limitations. The foals were too small for such an examination, and it was not possible to reach the right kidney in adult horses. However, numerous studies have indicated that transabdominal renal ultrasonography has similar repeatability and reproducibility to the transrectal examination [36,37]. A third potential limitation of this study was that the animals were only of three age groups. It would be useful to carry out a further analysis on a wider group of horses of different ages, which would improve accuracy of the correlation of the RI values with age. We believe that the assessment of the RI range should be complemented by a study of its values in horses with acute and chronic kidney failure in order to determine the usefulness of this parameter in the detection and differentiation of kidney disease. Based on our clinical experience during the Doppler measurements of the RI in healthy horses, we believe that considerable experience of the examiner is required to measure this index. Due to this and to lack of an additional portable ultrasound device, the authors of the study did not carry out the assessment of intra- and interobserver repeatability and variability. In addition, RI measuring method in horses is time-consuming and requires that the patient be constrained, which is not always possible, which is another study limitation. The clinical application of this method in non-cooperating animals remains questionable.

## Conclusion

The references value of the RI of intrarenal arteries in warmblood non-racing horses is similar to the values obtained in previous studies, although no significant differences in the RI between the left and right kidney was obtained, irrespective of the sex and age of the horses. However, the RI reference values were higher in elderly horses compared to foals and adult horses, which could be misinterpreted as increased values, suggesting kidney disease. The arterial pulse pressure should be measured when interpreting RI values, as it has a positive effect on them. The obtained results were highly repeatable. However, the assessment of RI may be difficult and time-consuming in uncooperative horses.
